# Supplementary material for: Confocal Laser Scanning Microscopy as a Method for Identifying Variation in Puparial Morphology and Establishing Characters for Taxonomic Determination
Source: Insects. 2025 Jan 16;16(1):88. doi: 10.3390/insects16010088 (PMC11766249; doi:10.3390/insects16010088)
Supplement: Supplementary file 1 [file insects-16-00088-s001.zip › supplementary.pdf]

# Supplemental Information: MACRO USED FOR QUANTIFICATION OF IMAGES

```
////////////////////////////////
// global variables
var orig = 0;//stores name of picture
var title1 = 0;//Name of Results Table

// for getTimeString() function
var TimeString = 0;//Date and Time for macro run

// for createTable() function
var f=0;//Result Table

// for getDateTime() function
var DateTime = 0;//Date and Time from Exif info

//for folderChoice() function
var savedir = 0;// Directory where files will be saved
var filecount = 0;// how many files are there?
var jpgcount = 0;// how many jpgs in the folder?
var folderstoprocess = newArray(1000000);//folder names to process
var filestoprocess = newArray(1000000);//file names to process
/////////////////////////////
```

```
///////////////////////////////
resetImageJ();
createTable(); // creates the results table
getTimeString(); // makes the timestring
print(TimeString);
// control structure of macro to allow in-determinate number of images to be processed.
folderChoice(); // run it first time
//set line width thicker so it's visible
run("Line Width...", "line=5");
do {
	choice = getBoolean("Do you want to process another folder?");
	if (choice==0) {
		start = getTime();
		folderstoprocess = Array.trim(folderstoprocess, filecount);
		filestoprocess = Array.trim(filestoprocess, filecount);

		for (z=0; z<filestoprocess.length; z++) {
	roiManager("reset");

	showProgress(z/filestoprocess.length);

	open(folderstoprocess[z]+filestoprocess[z]);

	orig = getTitle();

	selectWindow(orig);
```

```
// User selection horizon
// 4 = Line selection
run("Select None");//Selecting nothing just in case
setTool(4);//Line Tool

message= "Line Selection Required\n Please create a Horizon line";
waitForUser(message);

// get horizon information
getLine(x1, y1, x2, y2, lineWidth);

// set line color to yellow
run("Colors...", "foreground=magenta background=white selection=magenta");
// fill in line (need fill because thickness > 1)
run("Fill", "slice");

if (x1==-1) // if x1 is less than 0 then it is not a proper line
  exit("This macro requires a straight line selection");

dx = x2-x1; // change in x
dy = y2-y1; // change in y

slopehoriz = dy / dx;

length = sqrt(dx*dx+dy*dy);
print("Length of horizon:", length);
print("slope of horizon:", slopehoriz);

x3 = newArray(6);
x4 = newArray(6);
```

```
y3 = newArray(6);
y4 = newArray(6);

dxO = newArray(6);
dyO = newArray(6);

slopeobject = newArray(6);
lengthobject = newArray(6);
angleofobjecthoriz = newArray(6);

// set line color to yellow
run("Colors...", "foreground=yellow background=white selection=magenta");

// assumes 6 slits
for (i=0; i<6; i++) {
        run("Select None");//Selecting nothing just in case
        setTool("multipoint");//Line Tool

        message= "Select Two endpoints: oriface "+ i+1 + "of 6\n";
        waitForUser(message);

        // get horizon information
        getSelectionCoordinates(xpoints, ypoints);

        Array.print(xpoints);
        Array.print(ypoints);

        x3[i] = xpoints[0];
        y3[i] = ypoints[0];
        x4[i] = xpoints[1];
```

```
y4[i] = ypoints[1];

makeLine(x3[i], y3[i], x4[i], y4[i], 5);
// fill in line (need fill because thickness > 1)
run("Fill", "slice");

dxO[i] = x4[i]-x3[i]; // change in x
dyO[i] = y4[i]-y3[i]; // change in y

slopeobject[i] = dyO[i] / dxO[i];

lengthobject[i] = sqrt(dxO[i]*dxO[i] + dyO[i]*dyO[i]);
//print("Length of object:", lengthobject[i]);
//print("slope of object:", slopeobject[i]);
angleofobjecthoriz[i] = 180/PI * atan( (slopehoriz - slopeobject[i])/ (1+(slopehoriz *
slopeobject[i])));
//print("angle of object from horizon:", angleofobjecthoriz[i]);

if (i <3) {
    angleofobjecthoriz[i] = abs(angleofobjecthoriz[i]);
} else {
    angleofobjecthoriz[i] = 180- abs(angleofobjecthoriz[i]);
}
//Print results for each oriface
print(f, orig + "\t" + i+1 + "\t" + lengthobject[i] + "\t" + angleofobjecthoriz[i] + "\t" + x3[i] + "\t" +
y3[i] + "\t" + x4[i] + "\t" + y4[i]);

}

selectWindow(orig);
//save with false color
```

```
            saveAs("jpg", savedir + orig + "_Painted");

            run("Select None");

            //reset ROIs for netting/stripes/shell diagnostics
            roiManager("reset");

            // close unnecessary image windows
            run("Close All");

            selectWindow("Results Table (results in px)");
            saveAs("Text", savedir + "OrifaceResults.temp");

                  } // end of image loop

            } // end of if statement when choice ==0
            if (choice==1) {
            folderChoice();
            }
} while (choice==1);
exit("Finished!");
////////////////////////////////////////////////////////////////////////////
//These are the functions called by the macro
////////////////////////////////////////////////////////////////////////////
function getTimeString() {
// time string for folder name

MonthNames = newArray("Jan","Feb","Mar","Apr","May","Jun","Jul","Aug","Sep","Oct","Nov","Dec");
  DayNames = newArray("Sun", "Mon","Tue","Wed","Thu","Fri","Sat");
  getDateAndTime(year, month, dayOfWeek, dayOfMonth, hour, minute, second, msec);
```

```
    TimeString = DayNames[dayOfWeek]+"_";
    if (dayOfMonth<10) {TimeString = TimeString+"0";}
    TimeString = TimeString+dayOfMonth+"_"+MonthNames[month]+"_"+year+"_";
    if (hour<10) {TimeString = TimeString+"0";}
    TimeString = TimeString+hour+"";
    if (minute<10) {TimeString = TimeString+"0";}
    TimeString = TimeString+minute+"_";
    if (second<10) {TimeString = TimeString+"0";}
    TimeString = TimeString+second;

} // end of getTimeString()
//////////////////////////////////////////
//////////////////////////////////////////
function folderChoice() {

            path = getDirectory("Choose a folder of images to analyze");
            print(path);
            // saves a folder for the processed images at your path with the following name
            savedir = path+TimeString+File.separator;
            File.makeDirectory(savedir);

            filelist = getFileList(path);
   for (z=0; z<filelist.length; z++) {
                        if (endsWith(filelist[z],"JPG")) {
            folderstoprocess[filecount] = path;
            filestoprocess[filecount] = filelist[z];
            jpgcount++;
            filecount++;
                        }
                        if (endsWith(filelist[z],"jpg")) {
```

```
folderstoprocess[filecount] = path;
filestoprocess[filecount] = filelist[z];
jpgcount++;
filecount++;
            }
            if (endsWith(filelist[z],"BMP")) {
folderstoprocess[filecount] = path;
filestoprocess[filecount] = filelist[z];
jpgcount++;
filecount++;
            }
            if (endsWith(filelist[z],"bmp")) {
folderstoprocess[filecount] = path;
filestoprocess[filecount] = filelist[z];
jpgcount++;
filecount++;
            }
            if (endsWith(filelist[z],"tif")) {
folderstoprocess[filecount] = path;
filestoprocess[filecount] = filelist[z];
jpgcount++;
filecount++;
            }
            if (endsWith(filelist[z],"TIF")) {
folderstoprocess[filecount] = path;
filestoprocess[filecount] = filelist[z];
jpgcount++;
filecount++;
            }
            if (endsWith(filelist[z],"tiff")) {
```

```
            folderstoprocess[filecount] = path;
            filestoprocess[filecount] = filelist[z];
            jpgcount++;
            filecount++;
                        }
                        if (endsWith(filelist[z],"TIFF")) {
            folderstoprocess[filecount] = path;
            filestoprocess[filecount] = filelist[z];
            jpgcount++;
            filecount++;
                        }
            }
var count = (count+1);
}// end of Folderchoice Function
 //////////////////////////////////////
//////////////////////////////////////
function createTable() {
// creates a custom results table or clears the current open if open
  title1 = "Results Table (results in px)";
  title2 = "["+title1+"]";
  f = title2;
  if (isOpen(title1))
     print(f, "\\Clear");
  else
     run("Table...", "name="+title2+" width=900 height=300");
  print(f, "\\Headings:File\trep\tlength\tangle\txstart\tystart\txstop\tystop");
} // end of createTable()
////////////////////////////////////////

//////////////////////////////////////
```

```
function clearScale() {

run("Set Scale...", "distance=0 known=0 pixel=1 unit=pixel global");

} // end of ClearScale () function {

//////////////////////////////////////

//////////////////////////////////////

function resetImageJ() {


	requires("1.48d");

	// Only if needed uncomment these lines

	//run("Memory & Threads...", "maximum=1500 parallel=4 run");

	run("Options...", "iterations=1 count=1 edm=Overwrite");

	run("Line Width...", "line=1");

	run("Colors...", "foreground=black background=white selection=yellow");

	run("Clear Results");

	run("Close All");

	print("\\Clear");

	run("ROI Manager...");

	run("Input/Output...", "jpeg=75 gif=-1 file=.csv use_file copy_row save_column save_row");

	run("Set Measurements...", "area mean standard modal min centroid center perimeter bounding fit shape
feret's integrated median skewness kurtosis area_fraction stack redirect=None decimal=3");

}

//////////////////////////////////////
```
